# Supplementary figures and images for: Accumulation and transfer of polystyrene microplastics in Solanum nigrum seedlings
Source: PeerJ. 2023 Aug 31;11:e15967. doi: 10.7717/peerj.15967 (PMC10475273; doi:10.7717/peerj.15967)

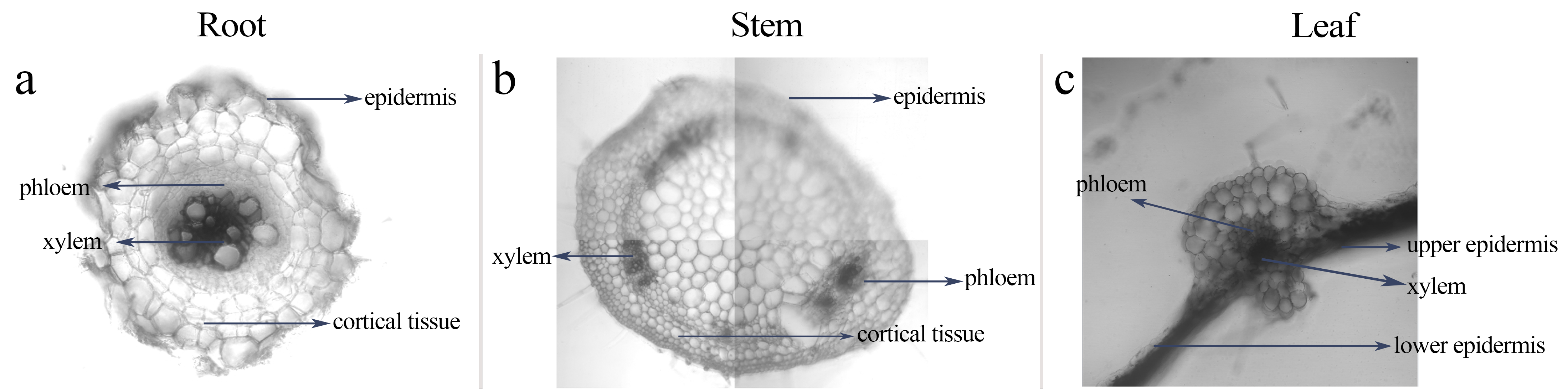

Supplement: Supplemental Information 1 [file peerj-11-15967-s001.png]
